# Three-month outcomes and cost-effectiveness of interferon gamma-1b in critically ill patients: a secondary analysis of the PREV-HAP trial

**DOI:** 10.1186/s40560-024-00753-z

**Published:** 2024-10-11

**Authors:** Marwan Bouras, Philippe Tessier, Cécile Poulain, Solène Schirr-Bonnans, Antoine Roquilly

**Affiliations:** 1 Nantes Université, CHU Nantes, Service d’Anesthésie Réanimation, 44000 Nantes, France; 2 Center for Research in Transplantation and Translational Immunology, UMR 1064, Nantes Université, Inserm, CHU Nantes, 44000 Nantes, France; 3 Department of Anaesthesia, Intensive Care Medicine and Peri-Operative Medicine, Hôpital de La Cavale Blanche, Bd Tanguy Prigent, CHRU de Brest, 29200 Brest, France; 4 SPHERE, Service Evaluation Economique Et Développement Des Produits de Santé, Direction de La Recherche Et de LInnovation, Nantes Université, INSERM, MethodS in Patients-Centered Outcomes and HEalth Research, Université́, CHU Nantes, 44000 Nantes, Nantes France; 5 Department of Microbiology and Immunology, University of Melbourne, Peter Doherty Institute for Infection and Immunity, Melbourne, VIC Australia

**Keywords:** Critical illness-related immunosuppression, Cost-effectiveness analysis, Interferon gamma‑1b, Quality-Adjusted Life-Years

## Abstract

**Background:**

Interferon gamma‑1b has been proposed to treat critical illness-induced immunosuppression. We aimed to determine the effects on 90-day outcomes and the cost-effectiveness of interferon gamma‑1b compared to placebo in mechanically ventilated critically ill patients.

**Methods:**

A cost-effectiveness analysis (CEA) was embedded in the “PREV-HAP trial”, a multicenter, placebo‑controlled, randomized trial, which randomly assigned critically ill adults under mechanical ventilation to receive interferon gamma or placebo. The CEA compared interferon-gamma with placebo using a collective perspective at a 90-day time horizon. The primary outcome was the incremental cost-effectiveness ratio (ICER) expressed in terms of adjusted cost per adjusted Quality-Adjusted Life-Years (QALYs) gained. QALYs were estimated from the responses of patients and proxy respondents to the health-related quality of life questionnaire EQ-5D-3L.

**Results:**

The 109 patients in the PREV-HAP trial were included in the CEA. At day 90, all-cause mortality rates were 23.6% in the interferon group and 25% in the placebo group (Odds Ratio (OR) = 0.88 (0.40 –1.93) p = 0.67). The difference in the mean adjusted costs per patient at 90 days was €-1.638 (95%CI €-17.534 to €11.968) in favor of interferon gamma-1b. The mean difference in adjusted QALYs between interferon gamma-1b and the placebo group was + 0.019 (95%CI -0.005 to 0.043). The probability that interferon gamma-1b was cost-effective ranged from 0.60 to 0.71 for a willingness to pay a QALY between €20k and €150k for the base case analysis.

**Conclusion:**

Early administration of interferon gamma might be cost-effective in critically ill patients supporting the realization of other studies on this treatment. However, the generalization of the findings should be considered cautiously, given the small sample size due to the premature end of PREV-HAP.

*Trial registration* ClinicalTrials.gov Identifier: NCT04793568, Registration date: 2021–02-24.

**Supplementary Information:**

The online version contains supplementary material available at 10.1186/s40560-024-00753-z.

## Background

Patients admitted to the intensive care unit (ICU) for sepsis or trauma develop a systemic inflammatory response syndrome (SIRS) induced by the activation of innate immunity [1]. The subsequent response, called “critical illness-related immunosuppression”, is a physiological response inducing immunosuppression and immune tolerance to limit an overwhelming SIRS and promote tissue healing [2, 3]. This state of immune tolerance is a complex process involving innate immune cells via a decreased production of proinflammatory cytokines (interferon-gamma, Tumor Necrosis Factor-alpha), a reduced antigen presentation capacity (via monocyte Human Leukocyte Antigen-DR expression) and a reorganization of the neuro-hormonal systems (sympathetic, parasympathetic, hypothalamic-pituitary axis). The involvement of critical illness-related immunosuppression as a risk factor for hospital-acquired infections is well described in ICU patients [4] and its presence is associated with a risk of death of at least 50% in sepsis patients [5]. This immune reprogramming lasts for months after ICU discharge [6, 7] and is associated with long-term complications, including secondary infections, cardiovascular diseases, neurocognitive deficits and altered quality of life [8–10]. Thus, it has been proposed that treatments of critical illness-related immunosuppression can potentially decrease mortality and enhance the long-term outcomes of patients [11].

Interferon gamma-1b treatment has been used in several case reports of protracted infections in ICU patients. However, prior to our study, no randomized clinical trials had tested the treatment effects on in-ICU and post-ICU outcomes. We conducted the PREV-HAP placebo-controlled randomized trial to investigate the effects of human recombinant interferon gamma 1b (rHuIFN-γ, Imukin) treatment on hospital-acquired infections and post-ICU outcomes. [12]. Critically ill adults with acute organ failure on mechanical ventilation received either interferon gamma-1b or placebo, with the primary composite outcome being the occurrence of hospital-acquired pneumonia or all-cause mortality by day 28. The scientific rationale of the PREV-HAP trial was to restore immune competency in patients with critical illness related immunosuppression. While the study was discontinued early due to safety concerns related to respiratory complications (more hospital-acquired pneumonia (HAP) in the interferon group: RR 2.06, 95% CI 0.92–4.57), interferon gamma-1b differently affected early patient-centered outcomes, as in-ICU mortality tended to be lower in the treatment group (RR 0.68, 95% CI 0.25–1.85). The description of the long-term efficacy of interferon gamma-1b treatment for critical-illness-related immune suppression remains a significant point of interest in the field.

The effects of new immunotherapies in critical care, particularly their long-term outcomes, require thorough exploration to understand their potential benefits and limitations. A secondary analysis of the PREV-HAP trial is therefore indicated and offers an opportunity to provide initial insights into the efficacy and efficiency of interferon gamma-1b. Preliminary findings on QALYs and the cost-effectiveness of the intervention are valuable in guiding subsequent studies, either by supporting the continued exploration of interferon gamma-1b as a therapeutic option or by tempering the enthusiasm for its use. Indeed, several randomized clinical trials are still investigating the effects of interferon-gamma 1b in septic patients [13] or during hospital-acquired pneumonia (clinicaltrial.gov NCT05843786).

We thus hypothesized that the treatment could induce early and transient adverse events but benefit quality of life and reduce health care consumption in a long-term perspective. To respond to this question, we performed a cost-effectiveness analysis comparing interferon gamma-1b treatment with placebo in critically ill patients.

## Methods

This was a pre-planned ancillary analysis of the Prev-HAP trial [12]. The PREV-HAP study was a multicenter, parallel-group, double-blind, randomized trial designed to investigate the effects of interferon gamma-1b in critically ill patients at risk of hospital-acquired pneumonia. Briefly, patients aged between 18 and 85 years receiving invasive mechanical ventilation were eligible if presenting with one or more acute organ failures at the time of inclusion. Participants received five subcutaneous injections of 100 μg of recombinant interferon gamma-1b (interferon-gamma group) or matching placebo (placebo group). Patients were followed until day 90, and the rates of the primary outcome components (all-cause mortality at day 28 and 90 and HAP at day 28), mechanical ventilation-free days on day 90 were recorded. We also collected responses to the EQ-5D-3L questionnaire on day 28 and day 90 to estimate Quality-Adjusted Life Years (QALYs) for this ancillary cost-effectiveness analysis.

The cost-effectiveness analysis adopted a collective perspective including “all of the people or institutions affected (in terms of health effects or cost) by the production of an intervention within the scope of the overall patient care” thus excluding production losses from the base case analysis, as recommended by the French guidelines for the economic evaluation of health care programmes [14], and a 90-day time horizon. The costs, effectiveness and incremental cost-effectiveness ratio (ICER) comparing interferon gamma‑1b use against placebo to prevent HAP were estimated from individual patient data collected during the PREV-HAP trial. The ICER is defined as follows:

$${ICER} = ({C}_{I}-{C}_{P})/({E}_{I}-{E}_{P})$$

where ${C}_{I}, {C}_{P},{E}_{I},{E}_{p}$ correspond to estimates of total mean costs and total mean QALYs per patient over three months for the interferon gamma‑1b and the placebo arm, respectively.

### Ethics

The Ethics Committee of Ouest II Angers (France) approved the study protocol in March 2021 (N°2020–000620-18). This trial complied with the Declaration of Helsinki and was registered in March 2021 (number ClinicalTrial.gov NCT04793568). The patient’s legal surrogate provided written informed consent for participation.

### Outcomes: quality-adjusted life years (QALYs)

The outcomes were expressed in terms of QALYs assessed from EQ-5D quality of life measures at baseline, 28 and 90 days. QALYs combine information on length and health-related quality of life (HRQol) in a single index. They are estimated by weighting each period of time by an HRQol weight, called a utility value or score, where 0 represents being dead and 1 represents the best imaginable health state and such that a higher score corresponds to a more preferred health state (negative utility values are allowed for states considered worse than being dead). A year lived in perfect health thus represents one QALY, and three months lived in perfect health 0.25 QALYs. Utility values were determined from patient responses to the generic HRQol EQ-5D-3L questionnaire. The EQ-5D asked five questions about five dimensions of HRQol (mobility, self-care, usual activities, pain/discomfort and anxiety/depression). They can be answered using three ordered items ranging from 'no problem' to 'extreme/severe problems' with the dimension considered. The patient's responses to the EQ-5D questionnaire were converted to utility values using published tariffs for France [15]. At the beginning of the study, all patients were sedated and assigned a unique negative utility value of -0.402, which corresponds to the state of 'being unconscious' in the original EQ-5D-3L UK study [16]. As in previous studies [17, 18], when patients could not respond to the questionnaire on day 28 and/or day 90 due to their health condition, a proxy response was questioned. The proxy respondent was either a relative or a healthcare professional. The total number of QALYs over the three-month period corresponds to the areas under the curves obtained by applying linear interpolation between each EQ-5D utility scores [19]. Due to the short time horizon, QALYs were not discounted.

#### Resource use and costs estimation

Costs were estimated from a collective perspective. Resource use was documented using electronic case report forms (e-CRFs) for the initial hospitalisation and self-administered questionnaires for the follow-up period. The questionnaires asked participants retrospectively about their use of outpatient (GP and specialist consultations, antibiotic prescriptions, nurse visits) and hospital (rehospitalisation, emergency department visits and rehabilitation hospital stays) healthcare resources since their initial hospital discharge. They also reported on their use of some medical equipment (wheelchair, hospital bed), the help they received from relatives or professionals in carrying out their usual activities, and their absence from work. Resource units were valued monetarily using national health insurance tariffs, information from the national hospital cost database and wage information from the National Statistical Institute (Appendix 3). Total cost estimates over three months were obtained by multiplying resource quantities by their corresponding monetary value. All costs were expressed in 2019 euros. As with QALYs, no discounting was applied.

#### Missing data and multiple imputation

e-Table 1 indicates the percentage of missing observations per item. It ranges from 0% (initial hospital length of stay) to 24% (e.g. GP visits). The proportion of missing data is fairly balanced between the two arms with some exceptions such as the number of EQ-5D measures. We handled missing data for both costs and QALYs (derived from EQ-5D questionnaires) by using multiple imputation. Specifically, we used chained equations combined with predictive mean matching, which helps address the non-normal distribution of the data [20]. The regression models for imputation included the following baseline variables, which we assume are related to the missingness mechanism, costs, and EQ-5D scores: age, sex, whether patients were septic, whether they have trauma, a kidney, neurological, respiratory, or hemodynamic failure, respectively, SAPS (Simplified Acute Physiology Score) II score [21] and the study arm. We used 45 imputation sets. All analyses were performed using Stata version 15.0 (StataCorp, College Station, Texas 77,845 USA).

#### Base case cost-effectiveness analysis

The base case analysis was performed according to an intention-to-treat (ITT) principle, used imputed missing information and estimated adjusted differences in costs and QALYs. Patients were kept in the arm to which they were assigned whether they received interferon gamma‑1b or not and whether they received the expected dose or not. Differences in costs and QALYs were estimated using seemingly unrelated regression which accounts for the correlation between costs and QALYs [20]. The two regression equations for costs and QALYs contained the following explanatory variables: age, sex, whether the patients were septic, admission for trauma, a kidney, a neurological, a respiratory, or a hemodynamic failure, respectively, and the study arm. QALYs were also adjusted for the SAPS II score. Because costs and QALYs have non-normal distributions, the confidence intervals around their respective differences between the two arms were estimated by bootstrapping the regressions (1,000 replications) using bias-corrected and accelerated bootstrapping [22]. Estimates were used to calculate the adjusted mean ICER [23] (the ratio of the difference in total mean costs divided by the difference in mean QALYs) and the corresponding adjusted incremental net monetary benefit (the difference between monetized QALYs and costs). To take into account sampling uncertainty, the results were presented as an acceptability curve plotting the probability that interferon gamma‑1b was cost effective compared to placebo for different threshold monetary values for a QALY: €20,000, €50,000, €100,000 and €150,000, respectively.

#### Sensitivity analyses

We conducted several sensitivity analyses to test the robustness of our results. First, we analyzed the complete case sample, which included only patients with no missing data. Second, we explored the possibility that data were not missing at random, an assumption required for multiple imputation. In this scenario, we assumed that patients with missing data had a lower quality of life (25% below the imputed EQ-5D utility value) and higher costs (25% above the imputed cost). Third, we ran an analysis using a baseline EQ-5D utility value of zero, instead of -0.402, to estimate QALYs. Fourth, we performed an analysis from a societal perspective that included production losses calculated using the human capital method. Finally, we conducted an analysis without adjusting for baseline covariates, except for the SAPS-II score in the equation for QALYs.

## Results

### Study population

From April 2021 through October 2021, 109 patients underwent randomization and 108 completed the follow-up. (See the flow chart in e-Fig. 1.). All-cause mortality at day 28 was 12.7% in the interferon gamma group and 17% in the placebo group (odds ratio (OR) = 0.68, 95% CI: 0.25–1.85, p = 0.60). By day 90, mortality was 23.6% in the interferon group and 25% in the placebo group (OR = 0.88, 95% CI: 0.40–1.93, p = 0.67). The median duration of ICU stay was 18 days (interquartile range (IQR) 15–26) in the interferon group compared to 22 days (IQR 14–28) in the placebo group (OR = 0.95, 95% CI: 0.61–1.47, p = 0.82). The median duration of hospitalization was 50 days (IQR 31–62) in the interferon group and 47 days (IQR 36–63) in the placebo group (OR = 1.19, 95% CI: 0.76–1.88, p = 0.83). Neurological failure at ICU admission was the most frequent organ failure, reported in 84% of patients. The main characteristics at baseline are reported in Table 1.

**Table 1:** Baseline characteristics of participants

	Interferon gamma group (n=55)	Placebo group (n=53)
Age, years, median (25–75th percentile)	57 (41–64)	58 (44–67)
Sex, Male: n/N (%)	34/55 (61.8)	38/54 (70.4)
Body Mass Index, kg/m2, median (25–75th percentile)	25 (22–29)	27 (23–31)
Comorbidities, n/N (%)		
Cardiac insufficiency (NYHA > 2)	1/55 (1.8)	3/54 (5.6)
Active smoking	15/55 (27.3)	19/54 (35.2)
Diabetes mellitus	5/55 (9.1)	6/54 (11.1)
SAPSII, median (25–75th percentile)	45 (37–54)	45 (37–52)
SOFA, median (25–75th percentile)	7 (5–10)	7 (5–9)
Cause of hospitalization in ICU, n/N (%)		
Trauma	27/55 (49.1)	24/54 (44.4)
Surgical	17/55 (30.9)	20/54 (37)
Sepsis	2/55 (3.6)	4/54 (7.4)
Other	9/55 (16.4)	6/54 (11.1)
Organ failure at inclusion, yes, n/N (%)		
Neurological	48/55 (87.3)	43/54 (79.6)
Hemodynamic	20/55 (36.4)	15/54 (27.8)
Respiratory	7/55 (12.7)	8/54 (14.8)
Kidney	3/55 (5.5)	4/54 (7.4)

SAPSII = Simplified Acute Physiology Score II, SOFA : Sequential Organ Failure Assessment

### Outcomes: quality-adjusted life-years (QALYs)

**Fig. 1:**
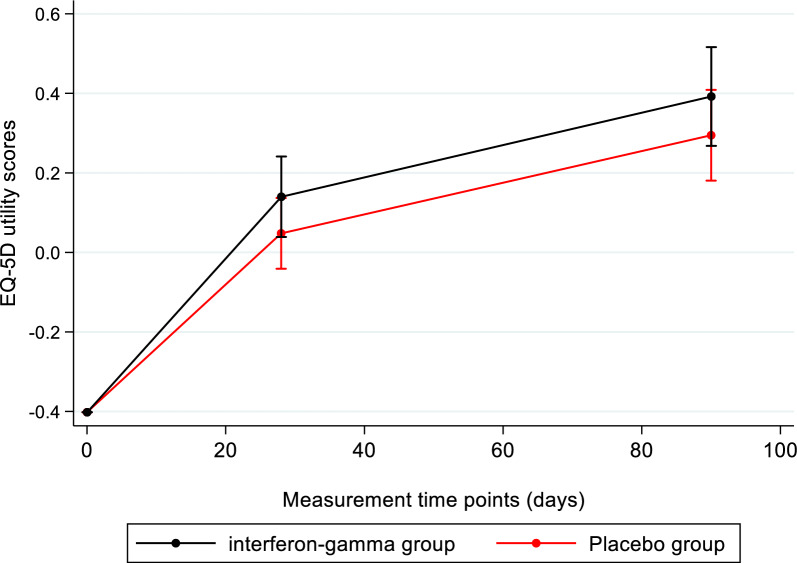
** Unadjusted EQ-5D utility scores.** At the beginning of the study, all patients were sedated and where assigned a unique negative utility value −0.402

The unadjusted EQ-5D utility scores are plotted in Fig. 1.

At baseline, the score was assumed to be the same for all patients under invasive mechanical ventilation. On days 28 and 90, more proxy respondents were required to fill out the EQ-5D questionnaires in place of the patients in the placebo group than in the interferon-gamma group (e-Table 2). The mean EQ-5D scores mixing responses from patients and proxy respondents were 0.140 (95% CI 0.039 to 0.241) and 0.392 (95% CI 0.268 to 0.516) in the interferon gamma‑1b group and 0.048 (95% CI -0.041 to 0.137) and 0.295 (95% CI 0.181 to 0.409) in the placebo group for day 28 and day 90 respectively. The mean unadjusted number of QALYs that corresponds to the areas under the curves in Fig. 1 was therefore higher in the interferon gamma arm.

### Healthcare resource use and costs

e-Table 3 shows the unadjusted mean health care resource used per arm, and Table 2 reports the corresponding mean costs per patient for the ITT analysis with missing information.

Unadjusted mean total costs per patient were higher in the interferon gamma‑1b arm, €69.574 (95% CI, €59.818 to €79.330) than in the placebo arm, €67.980 (95% CI, €55.999 to €79.961) (Table 2). As expected, hospital-related costs were the main drivers, with no statistical differences between the two study arms (60.928 euros in the interferon group versus 59.471 euros in the placebo group). There are some differences in the mean costs corresponding to resources used during the follow-up period (e.g. GP consultations, use of antibiotics, and help from a professional).

**Table 2:** Unadjusted mean costs (sample with imputed missing values) in euros 2019

Cost type	Interferon gamma 1b arm (95% CI)	Placebo arm (95% CI)
Initial hospitalisation		
Initial hospitalisation(outside ICU) (€)	18.828 (12.323 to 25.333)	20.215 (13.338 to 27.093)
Initial hospitalisation (ICU)(€)	41.276 (32.896 to 49,656)	39.256 (31.118 to 47.394)
Interferon gamma 1b (€)	824 (812 to 836)	0
Total for initial hospitalisation	60.928 (51.285 to 70.571)	59.471 (48.222 to 70.721)
Follow-up*		
Rehabilitation hospital (€)	5.980 (3.807 to 8.153)	6.395 (4.241 to 8.550)
Rehospitalisation (€)	2.342 (−236 to 4.921)	1.851 (−5 to 3.707)
Emergency visits (€)	10.9 (−1.6 to 23.3)	10.8 (−1.4 to 23.0)
GP consultations (€)	22.7 (7.5 to 37.9)	8.9 (1.3 to 16.5)
Specialist consultations (€)	56.7 (26.3 to 87.1)	62.9 (9.2 to 116.7)
Nurse visits (€)	91.2 (16.7 to 165.7)	124.9 (28.0 to 221.8)
Antibiotics (€)	1.4 (0 to 2.8)	7.1 (2.7 to 11.6)
Wheelchair (€)	25.3 (0.9 to 49.7)	27.1 (0.6 to 53.7)
Medical bed (€)	6.8 (−2.2 to 15.9)	7.1 (−2.7 to 16.8)
Help by a relative (€)	86.8 (31.6 to 142.1)	0
Help by a professional (€)	21.9 (−14.7 to 58.5)	13.7 (−3.2 to 30.7)
Production loss (€)	8.387 (5.077 to 11.698)	7.483 (4.265 to 10.701)
Total for follow-up	8.646 (5.256 to 12.036)	8.509 (5.698 to 11.320)
Total costs (€)	69.574 (59.818 to 79.330)	67.980 (55.999 to 79.961)

* Difference tests for each cost item cannot be performed because the statistical techniques that account for the skewness of the cost distributions and that adjust for potential baseline differences between the two arms are applied when estimating differences in total costs and QALYs

### Cost-effectiveness analysis

For the base case analysis, the adjusted cost difference between interferon gamma and placebo was € –1.638 (95%CI –17.534 to 11.968) in favor of interferon gamma (Table 3) also the confidence interval was wide, and the difference was not statistically significant.

The adjusted difference in QALYs was 0.0192 (95%CI –0.005 to 0.043).

**Fig. 2:**
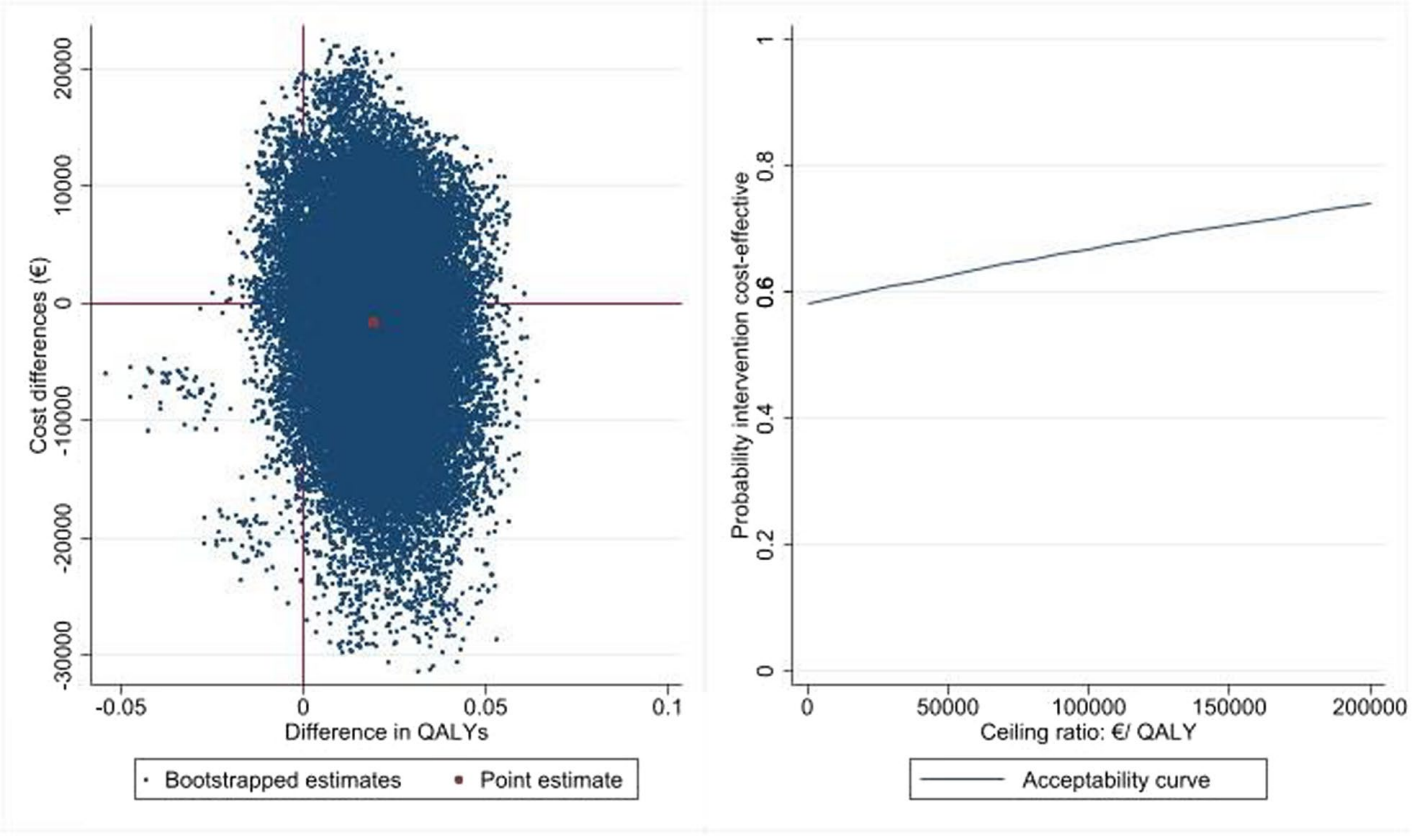
**Cost effectiveness plane (left) and acceptability curve (right)** **A**. Cost effectiveness plane (left) with adjusted costs and effects on baseline variables shows the distribution of 1000 bootstrap replications of differences in costs (y-axis) and effects (x-axis), for interferon gamma 1-b compared with placebo. **B**. Cost effectiveness acceptability curve (right) with adjusted costs and effects on baseline variables. The curve shows the probability that interferon gamma 1 bis cost effective or not (y-axis), depending on the willingness-to-pay per QALY (x-axis)

The 1,000 bootstrapped ICER estimates are plotted in Fig. 2A.

Most estimates (55%) fall in the southeast quadrant, indicating that interferon-gamma is associated with lower costs and better outcomes.

A therapy is considered cost-effective if the induced costs are lower than the maximum monetary amount society is willing to pay to gain one QALY (cost-effectiveness threshold or ceiling ratio expressed in €/QALY). The French Health Authority (HAS) does not provide a reference value for willingness-to-pay (WTP) for a QALY. Therefore, we estimated an acceptability curve that plotted the probability that interferon-gamma 1b was cost-effective for a wide range of WTP values. For the base case analysis, this probability was always above 50%, whatever the WTP for a QALY was, and it increased with the WTP value (Fig. 2B). It ranged from 60% (WTP/QALY €20.000) to 74% (WTP/QALY €150.000).

### Sensitivity analyses

We tested the robustness of this result through five sensitivity analyses (Table 3). Three produced results consistent with the main findings: a missing not at random scenario, a societal perspective analysis including production losses, and a scenario using an alternative EQ-5D baseline utility value. In these scenarios, the probability that interferon gamma could be cost-effective did not fall below 57% for a WTP for a QALY of €20.000, and it consistently increased with the WTP value (Table 3). Two scenarios produced different results: the analysis of complete case and the one without covariate adjustment. In the first case (sens. A), the probability that interferon gamma would be cost-effective ranged from 27% (WTP/QALY €20.000) to 37% (WTP/QALY €150.000). In the second case (sens E.), the ICER was estimated at €77.625, and the probability that interferon gamma would be cost-effective ranged from 44% (WTP/QALY €20.000) to 57% (WTP/QALY €150.000) (Table 3).

## Discussion

To our knowledge, this work is the first cost-effectiveness analysis evaluating the efficiency of a treatment of critical-illness-related induced immunosuppression. It suggests that interferon gamma-1b may be a dominant strategy compared to placebo, as it was associated with lower adjusted costs and a higher adjusted number of QALYs. However, the analyses were conducted on a reduced sample due to the early termination of the PREV-HAP trial**.** The difference in the mean number of QALYs may be due to a better recovery in terms of HRQol in the interferon-gamma arm (higher mean EQ-5D utility values on day 28 and day 90). Although this could reflect a true health difference between patients who were administered interferon gamma and those who received placebo, other explanations cannot be ruled out. One possible explanation might be a potential imbalance in baseline health status and severity [12]. The randomization process should have reduced this risk but it may still be a concern due to the premature end of the study. To limit this risk, we adjusted the analysis of QALYs differences on SAPS-II score. Another possible explanation may be that it is partly explained by the mix of patients and proxy respondents to the EQ-5D questionnaire, which we allowed to limit the amount of missing information. There may be concerns about the comparability of patients' and proxy respondents’ answers to the EQ-5D. However, several works suggest that their agreement level is good [24]. Specifically, some studies found that differences in EQ-5D utility values between patients and proxy respondents were small and not significant [25] and not susceptible to systematic bias [26]. Hence, the observed differences in EQ-5D utility scores could reflect a true difference in changes in HRQol between groups. This would explain why proxy answers were more frequently needed in the placebo arm than in the interferon-gamma 1b group at day 90.

While respiratory tolerance was poor in the PREV-HAP population, more QALYs were associated interferon-gamma-1b treatment. Neurological failure (coma) was the most frequent organ failure at the time of randomization, reported in 91 (83.5%) patients. It has been shown that neurons directly respond to interferon gamma‑derived from meningeal T cells to elevate tonic GABAergic inhibition and prevent aberrant hyper-excitability in the prefrontal cortex in mice [27]. These data suggest that social deficits in numerous neurological and psychiatric disorders may result from impaired circuitry homeostasis derived from dysfunctional immunity induced by interferon γ and that interferon gamma‑1b administration could improve neurological outcomes in patients [28]. The improvement in the EQ-5D score and the greater proportion of patients able to respond to the questionnaire at 90 days might reflect an interferon gamma‑1b-induced improvement in neurological status However, this hypothesis remains speculative and needs to be confirmed. It is also noteworthy that, the difference in costs between the two arms has wide confidence interval and appears to depend on whether the analysis was adjusted or not for stratification variables. Without adjustment, intervention group is associated with higher costs, mainly due to differences in-ICU length of stay and the administration of interferon gamma. However, after adjustment, the difference in costs was € 1.638, in favor of interferon gamma‑1b. This change between the raw and adjusted costs may be due to some imbalances in comorbidities between the two study arms [12]. The adjusted analysis was retained because it considers potential imbalances at baseline between the arms and adjusting for prognostic variables may increase the power and precision of the statistical analysis in randomized trials [29]. The adjustment variables were defined as they are known to be prognostic factors of outcome in ICU patients, and they were relevant in our analysis [30].

In our study, two scenarios led to different results than the base case analysis. In the complete case analysis, interferon-gamma did not appear to be cost-effective regardless of the WTP for a QALY. However, complete case analysis should be considered for informational purposes only, as it is known to be inefficient and does not respect the ITT principle when considering multiple time points [19]. In the absence of covariate adjustment (except for the SAPS-II score in the regression for QALYs), interferon gamma is no longer a dominant strategy. However, it is cost-effective for a willingness-to-pay of more than €77,625 per QALY. The French institutions did not provide a reference value for a QALY. A recent study estimated that this value could be between €120,000 and €201,000 per QALY [19]. Using the lower value of €100,000 per QALY in this sensitivity analysis, interferon gamma has a 52% probability of being cost-effective (Table 3). In addition, failure to adjust for potential baseline imbalances may lead to a biased ICER [20].

**Table 3:** Adjusted differences in costs and QALYs, ICER and probability of cost-effectiveness

				Probability Interferon gamma 1b was cost-effective based on the range of willingness to pay (WTP) a QALY monetary values			
Analysis	Δcosts euros 2019 95% CI	ΔQALYs 95% CI	ICER	WTP = k€ 20	WTP = k€ 50	WTP = k€ 100	WTP = k€ 150
Adjusted^Ψ^ base case	− €1.638 (−17.534 to 11.968)	0.0192 (−0.005 to 0.043)	Dominant	0.60	0.63	0.67	0.71
Sens. A	€ 5.038 (−10.290 to 20.366)	0.0151 (−0.138 to 0.044)	333,028	0.27	0.30	0.34	0.37
Sens. B	− €2.216 (−18.215 to 11.678)	0.0168 (−0.005 to 0.039)	Dominant	0.62	0.65	0.68	0.71
Sens. C	−€1.112 (−17.987 to 14.228)	0.0192 (−0.005 to 0.043)	Dominant	0.57	0.59	0.63	0.67
Sens. D	−€1,638 (−17.534 to 11.968)	0.0192 (−0.005 to 0.043)	Dominant	0.60	0.63	0.67	0.71
Sens. E	€1,594 (−14,916 to 14,157)	0.0205 (−0.003 to 0.044)	77,625	0.44	0.47	0.52	0.57

QALY = Quality-Adjusted Life-Years estimated by EQ-5D-3L generic HRQol questionnaire

ICER = Incremental Cost-Effectiveness Ratio

Ψ: “Adjusted” on age, sex, whether the patients were septic, have a trauma, a kidney, a neurological, a respiratory, or a hemodynamic failure, the study arm for both costs and QALYs, and the SAPS-II for QALYs

* Note: the differences are estimated between Imukin and placebo so that a negative difference indicates that the placebo is associated with higher costs (resp. QALYs) compared to Imukin. Sensitivity analyses: A) complete cases analysis; B) Missing not at random scenario: imputed costs are inflated by 25% and imputed QALYs are decreased by 25%; C) Societal perspective including production losses (human capital approach); D) Use the value of 0 rather than -0.402 for the baseline EQ-5D value for all patients E) Similar as base case analysis but unadjusted (except for the SAPS-II score for QALYs)

Our study has several limitations. First, the number of patients in the two groups was reduced due to the early end of the PREV-HAP trial. The differences highlighted for QALYs, and costs were not statistically significant but provided interesting results demonstrating that interferon treatment does not lead to excess costs or over-utilization of healthcare resources and does not result in unfavourable long-term outcomes. Secondly, our analysis shows that the EQ-5D questionnaires was answered by proxies for roughly 50% of the population at day 28 and between 11 and 25% at day 90. While this data has been discussed above and could potentially be explained by better neurological recovery at day 90 in the interferon gamma-1b group, mixing patients and proxy respondents answers could possibly induce a comparability bias. However, this was the only approach available to limit the number of missing answers and it has been applied in previous cost-effectiveness analyses [17, 18]. Third, this cost-effectiveness analysis examines the impact of interferon-gamma administration at 3 months after randomization. While the majority of healthcare costs attributable to a stay in ICU are spent within 3 months, it is known that patients continue to experience significant quality-of-life improvements up to 1 year after their ICU stay. Some ICU complications, such as ICU-acquired weakness [31] or neurological recovery in traumatic brain-injured patients would require longer-term evaluation [32]. Fourth, although the PREV-HAP trial was designed to be an international randomized trial, all patients were included in France when the trial was prematurely ended. As healthcare systems organization varies between countries, the results of this cost-effectiveness analysis should be confirmed in other healthcare systems.

## Conclusion

In summary, this study suggests that interferon gamma-1b may be cost-effective compared to placebo for the treatment of critically illness-related immune suppression. However, interpretation and generalization of the findings should be approached with caution, given the small sample size due to the premature end of the PREV-HAP clinical trial and the combination of patient and proxy responses to the EQ-5D questionnaire.

## Supplementary Information

Additional file 1.

## Data Availability

The datasets used and/or analysed during the current study are available from the corresponding author on reasonable request.

## Abbreviations

CEA: Cost-effectiveness analysis
GP: General practitioner
HAP: Hospital-acquired pneumonia
ICER: Incremental cost-effectiveness ratio
ICU: Intensive care unit
IQR: Interquartile range
OR: Odds Ratio
QALY: Quality-Adjusted Life-Years
SAPS: Simplified Acute Physiology Score
SIRS: Systemic inflammatory response syndrome
WTP: Willingness-to-pay

## References

[1] Huber-Lang M, Lambris JD, Ward PA. Innate immune responses to trauma. Nat Immunol. 2018;19:327–41. 10.1038/s41590-018-0064-8. 29507356 10.1038/s41590-018-0064-8 PMC6027646

[2] Bouras M, Asehnoune K, Roquilly A. Immune modulation after traumatic brain injury. Front Med. 2022;9: 995044. 10.3389/fmed.2022.995044. 10.3389/fmed.2022.995044 PMC9751027 36530909

[3] Venet F, Monneret G. Advances in the understanding and treatment of sepsis-induced immunosuppression. Nat Rev Nephrol. 2018;14:121–37. 10.1038/nrneph.2017.165. 29225343 10.1038/nrneph.2017.165

[4] Hotchkiss RS, Monneret G, Payen D. Sepsis-induced immunosuppression: from cellular dysfunctions to immunotherapy. Nat Rev Immunol. 2013;13:862–74. 10.1038/nri3552. 24232462 10.1038/nri3552 PMC4077177

[5] Deutschman CS, Tracey KJ. Sepsis: current dogma and new perspectives. Immunity. 2014;40:463–75. 10.1016/j.immuni.2014.04.001. 24745331 10.1016/j.immuni.2014.04.001

[6] Tsai AS, Berry K, Beneyto MM, et al. A year-long immune profile of the systemic response in acute stroke survivors. Brain J Neurol. 2019;142:978–91. 10.1093/brain/awz022. 10.1093/brain/awz022 PMC6933508 30860258

[7] Roquilly A, Jacqueline C, Davieau M, et al. Alveolar macrophages are epigenetically altered after inflammation, leading to long-term lung immunoparalysis. Nat Immunol. 2020;21:636–48. 10.1038/s41590-020-0673-x. 32424365 10.1038/s41590-020-0673-x

[8] Kerckhoffs MC, Kosasi FFL, Soliman IW, et al. Determinants of self-reported unacceptable outcome of intensive care treatment 1 year after discharge. Intensive Care Med. 2019;45:806–14. 10.1007/s00134-019-05583-4. 30840124 10.1007/s00134-019-05583-4 PMC6534510

[9] Angriman F, Rosella LC, Lawler PR, et al. Sepsis hospitalization and risk of subsequent cardiovascular events in adults: a population-based matched cohort study. Intensive Care Med. 2022;48:448–57. 10.1007/s00134-022-06634-z. 35142896 10.1007/s00134-022-06634-z

[10] Taquet M, Skorniewska Z, Hampshire A, et al. Acute blood biomarker profiles predict cognitive deficits 6 and 12 months after COVID-19 hospitalization. Nat Med. 2023;29:2498–508. 10.1038/s41591-023-02525-y. 37653345 10.1038/s41591-023-02525-y PMC10579097

[11] Roquilly A, Torres A, Villadangos JA, et al. Pathophysiological role of respiratory dysbiosis in hospital-acquired pneumonia. Lancet Respir Med. 2019;7:710–20. 10.1016/S2213-2600(19)30140-7. 31182406 10.1016/S2213-2600(19)30140-7

[12] Roquilly A, Francois B, Huet O, et al (2023) Interferon gamma-1b for the prevention of hospital-acquired pneumonia in critically ill patients: a phase 2, placebo-controlled randomized clinical trial. Intensive Care Med 1–15. 10.1007/s00134-023-07065-0 10.1007/s00134-023-07065-0 PMC10112824 37072597

[13] Kotsaki A, Pickkers P, Bauer M, et al. ImmunoSep (Personalised Immunotherapy in Sepsis) international double-blind, double-dummy, placebo-controlled randomised clinical trial: study protocol. BMJ Open. 2022;12: e067251. 10.1136/bmjopen-2022-067251. 36600424 10.1136/bmjopen-2022-067251 PMC9772655

[14] Haute Autorité de Santé (2020) Choices in methods for economic evaluation

[15] Chevalier J, De Pouvourville G. Valuing EQ-5D using Time Trade-Off in France. Eur J Health Econ. 2013;14:57–66. 10.1007/s10198-011-0351-x. 21935715 10.1007/s10198-011-0351-x

[16] Dolan P, Gudex C, Kind P, Williams A. The time trade-off method: Results from a general population study. Health Econ. 1996;5:141–54. 10.1002/(SICI)1099-1050(199603)5:2%3c141::AID-HEC189%3e3.0.CO;2-N. 8733106 10.1002/(SICI)1099-1050(199603)5:2<141::AID-HEC189>3.0.CO;2-N

[17] Grieve R, Sadique Z, Gomes M, et al. An evaluation of the clinical and cost-effectiveness of alternative care locations for critically ill adult patients with acute traumatic brain injury. Br J Neurosurg. 2016;30:388–96. 10.3109/02688697.2016.1161166. 27188663 10.3109/02688697.2016.1161166

[18] Taylor C, Thompson K, Finfer S, et al. Hydroxyethyl starch versus saline for resuscitation of patients in intensive care: long-term outcomes and cost-effectiveness analysis of a cohort from CHEST. Lancet Respir Med. 2016;4:818–25. 10.1016/S2213-2600(16)30120-5. 27324967 10.1016/S2213-2600(16)30120-5

[19] Manca A, Hawkins N, Sculpher MJ. Estimating mean QALYs in trial-based cost-effectiveness analysis: the importance of controlling for baseline utility. Health Econ. 2005;14:487–96. 10.1002/hec.944. 15497198 10.1002/hec.944

[20] Faria R, Gomes M, Epstein D, White IR. A guide to handling missing data in cost-effectiveness analysis conducted within randomised controlled trials. Pharmacoeconomics. 2014;32:1157–70. 10.1007/s40273-014-0193-3. 25069632 10.1007/s40273-014-0193-3 PMC4244574

[21] Le Gall JR, Lemeshow S, Saulnier F. A new Simplified Acute Physiology Score (SAPS II) based on a European/North American multicenter study. JAMA. 1993;270:2957–63. 10.1001/jama.270.24.2957. 8254858 10.1001/jama.270.24.2957

[22] Mutubuki EN, El Alili M, Bosmans JE, et al. The statistical approach in trial-based economic evaluations matters: get your statistics together! BMC Health Serv Res. 2021;21:475. 10.1186/s12913-021-06513-1. 34011337 10.1186/s12913-021-06513-1 PMC8135982

[23] Bang H, Zhao H. Median-based incremental cost-effectiveness ratio (ICER). J Stat Theory Pract. 2012;6:428–42. 10.1080/15598608.2012.695571. 23441022 10.1080/15598608.2012.695571 PMC3577357

[24] Hernández JD, Spir MA, Payares K, et al. Assessment by proxy of the SF-36 and WHO-DAS 2.0. A systematic review. J Rehabil Med. 2023;55:4493. 10.2340/jrm.v55.4493. 37389563 10.2340/jrm.v55.4493 PMC10337773

[25] Pickard AS, Johnson JA, Feeny DH, et al. Agreement between patient and proxy assessments of health-related quality of life after stroke using the EQ-5D and Health Utilities Index. Stroke. 2004;35:607–12. 10.1161/01.STR.0000110984.91157.BD. 14726549 10.1161/01.STR.0000110984.91157.BD

[26] Gabbe BJ, Lyons RA, Sutherland AM, et al. Level of agreement between patient and proxy responses to the EQ-5D health questionnaire 12 months after injury. J Trauma Acute Care Surg. 2012;72:1102–5. 10.1097/TA.0b013e3182464503. 22491635 10.1097/TA.0b013e3182464503

[27] Filiano AJ, Xu Y, Tustison NJ, et al. Unexpected role of interferon-γ in regulating neuronal connectivity and social behaviour. Nature. 2016;535:425–9. 10.1038/nature18626. 27409813 10.1038/nature18626 PMC4961620

[28] Abd-El-Basset EM, Rao MS, Alsaqobi A. Interferon-gamma and interleukin-1Beta enhance the secretion of brain-derived neurotrophic factor and promotes the survival of cortical neurons in brain injury. Neurosci Insights. 2020;15:263310552094708. 10.1177/2633105520947081. 10.1177/2633105520947081 PMC7391446 32776009

[29] Kahan BC, Jairath V, Doré CJ, Morris TP. The risks and rewards of covariate adjustment in randomized trials: an assessment of 12 outcomes from 8 studies. Trials. 2014;15:139. 10.1186/1745-6215-15-139. 24755011 10.1186/1745-6215-15-139 PMC4022337

[30] Wilcox ME, Vaughan K, Chong CAKY, et al. Cost-Effectiveness Studies in the ICU: A Systematic Review*. Crit Care Med. 2019;47:1011–7. 10.1097/CCM.0000000000003768. 30985446 10.1097/CCM.0000000000003768

[31] Vanhorebeek I, Latronico N, Van den Berghe G. ICU-acquired weakness. Intensive Care Med. 2020;46:637–53. 10.1007/s00134-020-05944-4. 32076765 10.1007/s00134-020-05944-4 PMC7224132

[32] McCrea MA, Giacino JT, Barber J, et al. Functional outcomes over the first year after moderate to severe traumatic brain injury in the prospective Longitudinal TRACK-TBI Study. JAMA Neurol. 2021;78:982. 10.1001/jamaneurol.2021.2043. 34228047 10.1001/jamaneurol.2021.2043 PMC8261688

