# Supplementary material for: Three-month outcomes and cost-effectiveness of interferon gamma-1b in critically ill patients: a secondary analysis of the PREV-HAP trial
Source: J Intensive Care. 2024 Oct 11;12:40. doi: 10.1186/s40560-024-00753-z (PMC11468134; doi:10.1186/s40560-024-00753-z)
Supplement: Supplementary file 1 — Additional file 1. [file 40560_2024_753_MOESM1_ESM.docx]

**e-Table 1. Missing data per item and complete case sample**

| **Data** | **Percentage of missing*** | | |
| --- | --- | --- | --- |
|  | **Interferon gamma** | **Placebo** | **Total** |
| **Quality of life** |  |  |  |
| EQ-5D at day 28 | 20.0 | 14.8 | 17.4 |
| EQ-5D at day 90 | 20.0 | 16.7 | 18.4 |
| Total QALYs | 30.9 | 22.2 | 26.6 |
| **Resource use** |  |  |  |
| Hospitalisation stay (initial) outside ICU | 0 | 3.7 | 1.8 |
| Hospitalisation stay (initial) at ICU | 0 | 1.9 | 0.9 |
| GP visits | 18.2 | 24.1 | 21.1 |
| Specialist visits | 18.2 | 20.4 | 19.3 |
| Antibiotic use | 18.2 | 24.1 | 21.1 |
| Rehabilitation hospital stay | 18.2 | 18.5 | 18.4 |
| Rehospitalisation | 20.0 | 18.5 | 19.3 |
| Emergency visits | 18.2 | 18.5 | 18.4 |
| Nursing care | 21.8 | 22.2 | 22.0 |
| Professional help at home | 16.4 | 20.4 | 18.4 |
| Help from relative at home | 23.6 | 24.1 | 23.9 |
| Use of a wheelchair | 20.0 | 20.4 | 20.2 |
| Use of a medical bed | 21.8 | 24.1 | 22.9 |
| Production losses | 20.0 | 20.4 | 20.2 |
| At least one item missing | 42.0 | 38.9 | 40.4 |

**e-Figure 1. Trial flow chart.**

**203 assessed for eligibility**

Excluded (n= 94)

♦  Not meeting inclusion criteria (n= 56)

♦  Declined to participate (n= 18)

♦  Other reasons (n= 20)

**109 underwent Randomization**

**Allocation**

**55 assigned to Interferon group**

**54 assigned to placebo group**

**Follow-Up:**

**QUALY day 28 (n= 108)**

**53 included for follow-up at day 28**

- 7 patients died & 1 withdrawal.

**55 included for follow-up at day 28**

- 11 patients died

**Follow-Up:**

**QUALY day 90 (n= 108)**

**53 included for follow-up at day 90**

- 1 patient died

**55 available follow-ups at day 90**

**Analysis**

**e-Table 2: Breakdown of respondents to the EQ-5D questionnaire***

|  | Number, (Percentage) for available cases | |
| --- | --- | --- |
|  | Interferon gamma | Placebo |
| **Measurement at day 28**  Response by patient  Response by proxy | 25 (56.8)  19 (43.2) | 23 (50)  23 (50) |
| **Measurement at day 90**  Response by patient  Response by proxy | 39 (88.6)  5 (11.4) | 33 (73.3)  12 (26.7) |

* The baseline utility value was set to -0.402 for all patients so that there are no missing values at the baseline

**e-Table 3: Unadjusted mean resource count per arm**

| **Resource type** | **Interferon gamma**  **(95% CI)** | **n** | **Placebo**  **(95% CI)** | **n** |
| --- | --- | --- | --- | --- |
| **Hospital initial stay** |  |  |  |  |
| Hospitalisation not in ICU (day) | 17.7 (11.6 to 23.9) | 55 | 18.9 (12.5 to 25.4) | 52 |
| Hospitalisation ICU (day) | 21.5 (17.1 to 25.9) | 55 | 20.5 (16.2 to 24.7) | 53 |
| **Follow-up*** |  |  |  |  |
| Rehabilitation hospital (day) | 20.3 (13.3 to 27.2) | 45 | 22.2 (15.0 to 29.4) | 44 |
| Rehospitalisation (day) | 2.5 (-0.3 to 5.4) | 45 | 1.7 (0 to 3.4) | 44 |
| GP consultations (number) | 0.8 (0.2 to 1.4) | 45 | 0.4 (0 to 0.7) | 41 |
| Specialist consultations (number) | 2.0 (0.9 to 3.1) | 45 | 2.1 (0.1 to 4.0) | 43 |
| Nursing care (day) | 6.7 (1.0 to 12.4) | 44 | 8.1 (1.2 to 15.0) | 42 |
| Antibiotics (day) | 1.0 (0 to 2.0) | 46 | 4.0 (1.5 to 6.5) | 43 |
| Wheelchair (day) | 3.0 (-0.4 to 6.3) | 46 | 5.9 (0 to 11.9) | 44 |
| Medical bed (day) | 2.0 (-1.4 to 6.8) | 45 | 3.3 (-1.6 to 8.1) | 42 |
| Production loss (day) | 16.0 (6.2 to 25.9) | 46 | 10.4 (2.2 to 18.5) | 44 |

GP: general practitioner.
